# Study on Perforation Behavior of PTFE/Al Reactive Material Composite Jet Impacting Steel Target

**DOI:** 10.3390/ma16072715

**Published:** 2023-03-29

**Authors:** Hongda Li, Hui Duan, Zhili Zhang, Yuanfeng Zheng

**Affiliations:** 1 Missile Engineering Academy, Rocket Force Engineering University, Xi’an 710025, China; 2 State Key Laboratory of Explosion Science and Technology, Beijing Institute of Technology, Beijing 100081, China

**Keywords:** PTFE/Al, reactive material, shaped charge, composite jet

## Abstract

To study the penetration and cratering effect of reactive material composite jets, a series of experiments are carried out for the shaped charge (SC) with different composite liners damaging steel targets. The inner layer of composite liners is metal and the outer one is a polytetrafluoroethylene/aluminum (PTFE/Al) reactive material. Copper (Cu), titanium (Ti) and Al inner liners are used in this paper. The reactive material liner is composed of 73.5 wt.% PTFE and 26.5 wt.% Al powder through mass-matched ratios. Reactive material composite liners are prepared through machining, cold pressing and a sintering process. The SC mainly consists of a case, a composite liner, high-energy explosive and an initiator. The steel target is steel 45#, with a thickness of 66 mm. A standoff of 1.0 CD (charge diameter) is selected to conduct the penetration experiments. The experimental results show that when the inner layer of the composite liner is composed of Ti and Al, the hole diameters on the steel target formed by the reactive material composite jet are significantly larger than that of the inner Cu liner. By introducing the initiation delay time (*τ*) and detonation-like reaction model of PTFE/Al reactive materials, an integrated numerical simulation algorithm of the penetration and detonation-like effects of reactive material composite jets is realized. Numerical simulations demonstrate that the initial penetration holes on the steel targets are enlarged under the detonation-like effects of PTFE/Al reactive materials, and the simulated perforation sizes are in good agreement with the experimental results.

## 1. Introduction

Reactive materials have been one of the research hotspots in the field of new material and engineering applications in recent years. Commonly used reactive materials mainly include the mixture of a polymer powder matrix (such as PTFE) and certain amounts of energetic metal powders (such as metal, alloy, intermetallic compounds, etc.). Thus far, the formulation system of reactive materials mainly includes PTFE/Al, PTFE/Al/W (tungsten), PTFE/Ti/W, etc. [1,2,3]. They can be formed into various samples, such as reactive fragments, reactive liners and reactive rods, through the use of cold pressing and high-temperature sintering with special molds after being fully mixed [4,5,6,7,8]. These reactive material samples have metal-like strength and explosive-like energy properties, and they can release their chemical energy under intense dynamic loading or high pressure and high strain rates [9].

Generally, reactive liners prepared using a class of reactive materials can be applied to the SC (shaped charge). Under the action of the SC, the high-velocity reactive jet formed by the reactive liner can not only penetrate the target with its kinetic energy, but also enlarge the initial perforation or enhance the after-effect through the release of chemical energy. Due to their excellent performance, shaped charges with reactive liners have been studied extensively. Early in 2001, Baker [10] verified that reactive liners can effectively form a continuous jet similar to metal Al liners through X-ray tests. Li [11] studied the forming characteristics of PTFE/Ti/W reactive jets with SPH numerical simulations and pulsed X-ray experiments, and analyzed the expansion phenomenon in the process of reactive jet forming. Su [12] and Zheng [13] investigated the temperature and density evolution behaviors and distribution characteristics of PTFE/Al reactive jets. Guo [14] studied the influence mechanism of Al particle size on the forming characteristics and reaction degrees of PTFE/Al reactive jets. In terms of the reactive jets damaging designed targets, Daniels [15] and Xiao [16] conducted experiments on reactive jets penetrating concrete, and found that PTFE-based reactive jets had good explosion enhancement damage effects. Zheng [17] studied the damage behavior of PTFE/Al reactive jets impacting multilayer spaced targets, and the results showed that the damage effects of the Al plates mainly depended on the mass of the follow-through reactive materials. Guo [18] studied the combined damage effects of PTFE/Al reactive jets against the thickness of steel targets under different standoffs, and the theoretical analysis model of the reactive jet penetration was established by introducing the initiation delay time of the reactive materials. However, due to the limitations of the strength and ductility of PTFE/Al reactive materials, the penetration capability of the PTFE/Al reactive jet was significantly worse than that of traditional metal jets [19]. The penetration depth of the PTFE/Al reactive jet for the SC with a charge diameter of 66 mm ranged from 0.32 CD to 0.79 CD (charge diameter) [20]. The maximum penetration depth of the reactive liner shaped charge with a diameter of 46 mm to the C35 concrete target was only approximately 2.4 CD [21], which limited the application of this class of reactive materials in the SC to a certain extent.

To improve the penetration depth of the SC with the class of reactive materials, more attention has been paid to the composite liner formed due to the reactive material liner and the metal liner. Lee [22] fabricated composite liners by spraying Al or an Al/Ni alloy on the surface of copper liners, which can form a high-density copper jet that can penetrate targets; the energetic materials can then experience an exothermic reaction to improve the thermal effect inside of the target. Waddell [23] proposed a composite liner that fills the reactive liner between two inert liners, and the reactive materials can be mixed with a polymer-based powder or energetic metal composite powder. Xu [24] experimentally studied the penetration of the SC with PTFE/Al/W reactive material–Cu composite liners against steel 45#, and the results showed that the penetration depth caused by the composite jet was approximately 4.0 CD. Wang [25] studied the penetration performances of PTFE/Al reactive material–Cu jets under different liner thicknesses and standoffs, and the experimental results showed that the penetration depth of the reactive material–Cu jet on steel 45# could reach 3.7 CD. It could be seen that, compared with jets formed using traditional single-layer reactive material liners, the penetration depth of the reactive material–metal composite jet penetrating the steel target greatly increased. For the shaped charge, in addition to the penetration depth, the penetration hole diameter on the target formed by the jet is also an important index in engineering applications. However, not much research has been conducted on the perforation behavior of the SC with reactive material composite liners against steel targets, especially the influence of the inner metal liner material on the penetration hole diameters of reactive material composite jets.

In this paper, the penetration and cratering effects of the SC with reactive material composite liners against steel targets are investigated through static experiments and numerical simulations. Firstly, samples of reactive material–Cu liners, reactive material–Ti liners and reactive material–Al liners are fabricated. On this basis, penetration experiments of the SC with reactive material composite liners against steel targets are carried out. Then, the formation characteristics of the reactive material composite jets before impacting the target are investigated, including the formation morphology and performance parameters of the composite jets and the pressure and temperature distribution of the reactive materials. Finally, the combined cratering effects of the kinetic energy penetration and detonation-like reaction of the reactive material composite jets against steel targets are simulated and compared with the experimental results.

## 2. Penetration Experiments

### 2.1. Composite Liner Samples

**Figure 1:**
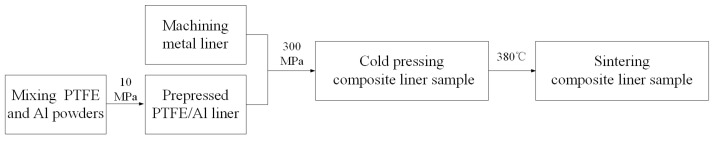
The flowchart of the reactive material composite liner preparatory process.

Composite liners consist of an inner layer metal liner and an outer layer PTFE/Al reactive material liner. The typical preparatory process of composite liners is shown in Figure 1, mainly divided into four steps [25]:

(1) The first step is processing the metal liner. The metal bar is processed using mechanical equipment and the shape of the metal liner is processed according to the drawing.
(2) The second step is preparing the PTFE/Al reactive materials. According to the formula design of the reactive materials, the PTFE powder and Al powder are crushed separately, and then mixed evenly with a special mixer. Generally, evenly mixed PTFE/Al compounds can be obtained through mixing for 30 min.
(3) The third step is cold pressing the composite liner. First, a certain amount of uniformly mixed PTFE/Al compounds is weighed out, poured into a special mold and the formed into the liner shape at a pressure of 10 MPa. Then, the metal liner is placed inside the prepressed PTFE/Al liner. At this time, a pressure of 300 MPa is used to press for 10 min. Before the sintering process, the cold-pressed composite liner is placed in a room temperature environment for several hours to remove residual stress in the liner.
(4) The fourth step is sintering the cold-pressed composite liner. The cold-pressed composite liner can be placed into a sintering furnace filled with nitrogen for high-temperature sintering. The maximum sintering temperature is 380 °C, and the temperature is kept for 4 h. Then, the temperature is reduced to 315 °C at 0.5 °C/min and maintained for 4 h. The sintered composite liner sample can then be cooled to room temperature while still in the furnace.

**Figure 2:**
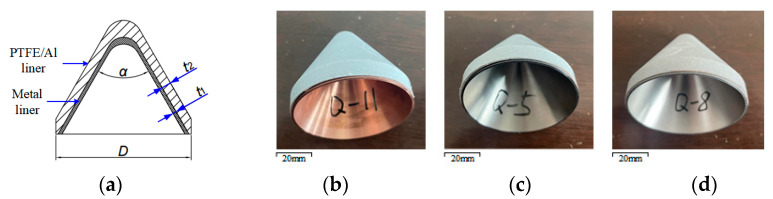
Schematic structure and sample of composite liner: (**a**) schematic structure; (**b**) reactive material–Cu liner; (**c**) reactive material–Ti liner; (**d**) reactive material–Al liner.

The schematic structure and samples of the reactive material composite liners are shown in Figure 2. In these experiments, the formula design of the reactive materials was composed of 73.5 wt.% PTFE and 26.5 wt.% Al powder. After the cold pressing and sintering processes, the density of the PTFE/Al liner was approximately 2.3 g/cm^3^. To study the effect of the inner metal liner material on the crater-expanding behavior of the steel target under the effect of the SC with the composite liner, three composite liners, reactive material–Cu liner, reactive material–Ti liner and reactive material–Al liner, were selected to carry out the penetration experiments. The wall thickness of the three types of composite liners was consistent to ensure the same mass of the main charge. The wall thickness of the metal liner *t*_1_ was 1 mm, and the PTFE/Al liner thickness *t*_2_ was 5 mm. The caliber diameter *D* and cone angle *α* of the composite liners were 66 mm and 60°, respectively.

### 2.2. Experimental Setup

**Figure 3:**
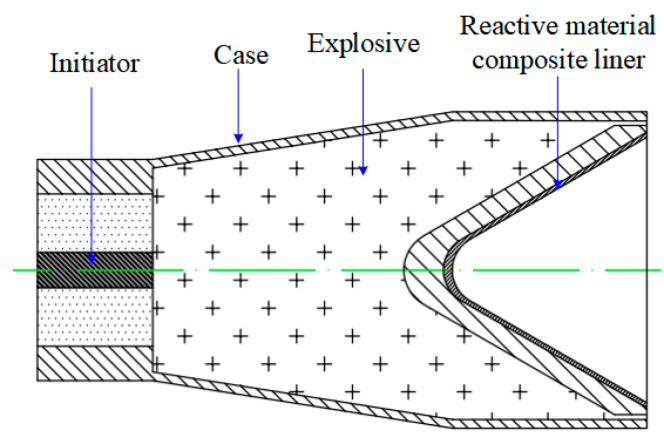
Schematic structure of the SC with the reactive material composite liner.

**Figure 4:**
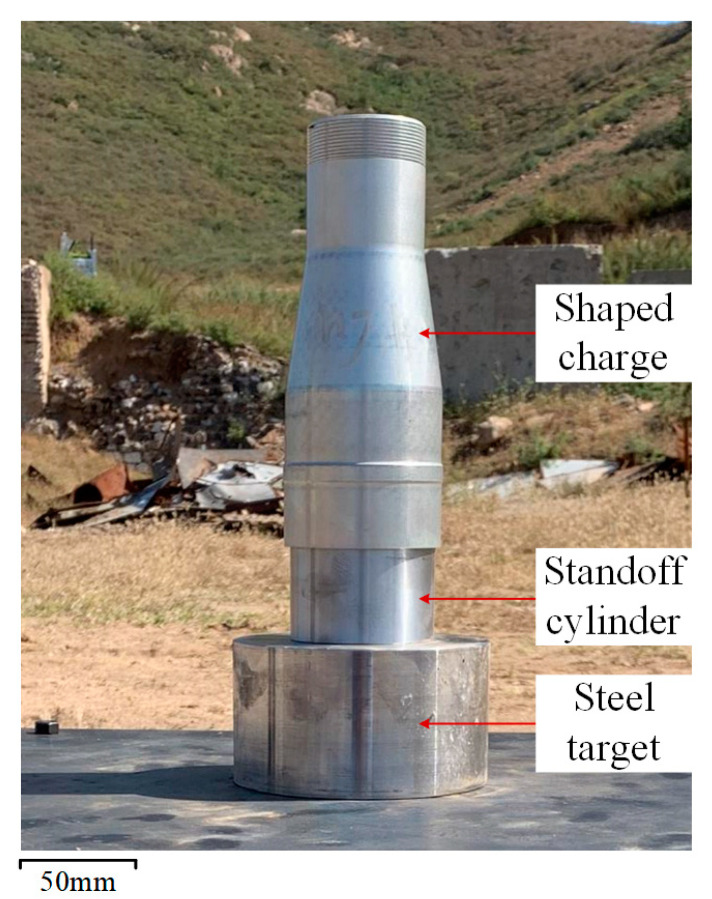
Experimental setup of the SC against steel target.

The schematic structure of the SC with the reactive material composite liners is shown in Figure 3. The SC mainly consisted of a case, a composite liner, high-energy explosive and an initiator. The length of the main charge was 110 mm and its diameter was 66 mm. The main charge was JH-2 high-energy explosive, which was detonated with an initiator placed in the center of the bottom of the SC. The material of the case was 2024 Al and its thickness was 2 mm. The experimental setup of the SC against the steel target is shown in Figure 4. In these experiments, the standoff cylinders were the same and their value was 1.0 CD (*H* = 66 mm). The standoff refers to the distance from the bottom of the SC to the surface of the target. A hollow Al cylinder with an outer diameter of 76 mm and an inner diameter of 64 mm was selected for the standoff. The steel target was steel 45# with a diameter of 120 mm and a height of 66 mm.

### 2.3. Experimental Results

**Figure 5:**
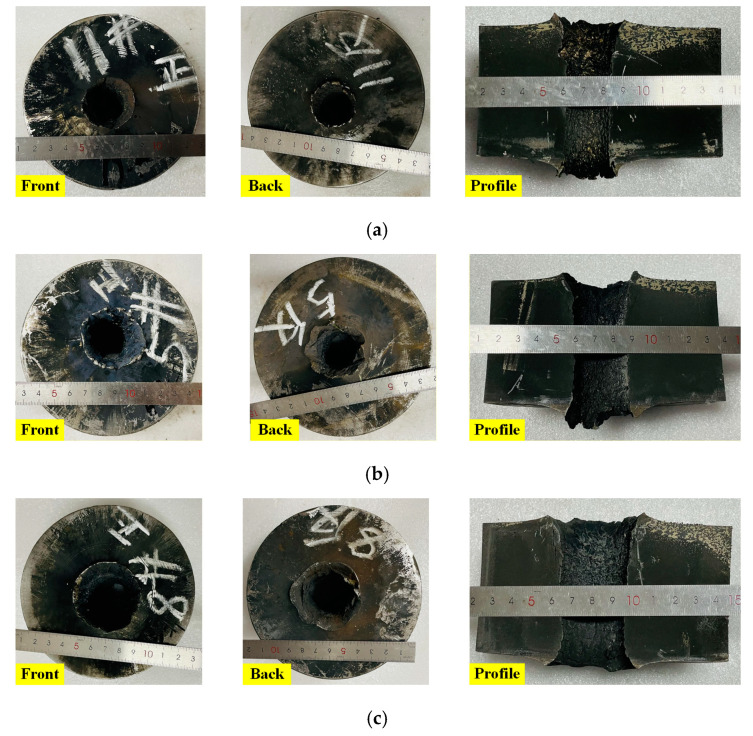
Perforation effects of the steel targets due to composite jets impact: (**a**) reactive material–Cu jet; (**b**) reactive material–Ti jet; (**c**) reactive material–Al jet.

The typical damage effects of the three kinds of SCs with the reactive material composite liners against the steel targets are shown in Figure 5. The experimental results showed that the three kinds of composite jets could penetrate the steel targets with a thickness of 1.0 CD. There were obvious petal bulge effects on the front and back craters of the steel targets. After the penetration experiments, the penetrated steel targets were divided with a special machine tool. It could be seen from the divided steel targets that the shapes of the through-hole formed by the three kinds of reactive material composite jets were similar, and that the diameters from the upper to the lower penetration craters were basically the same. There was a large amount of black product formed by the violent deflagration of reactive materials in the perforation channel and on the front and the back surface of penetrated steel targets. However, it can also be seen in Figure 5 that the through-hole diameters formed by the three kinds of reactive material composite jets were significantly different. Compared with the reactive material–Cu jet, the through-hole diameters were significantly larger under the combined action of the reactive material–Al jet and reactive material–Ti jet penetrating and deflagration effects.

**Figure 6:**
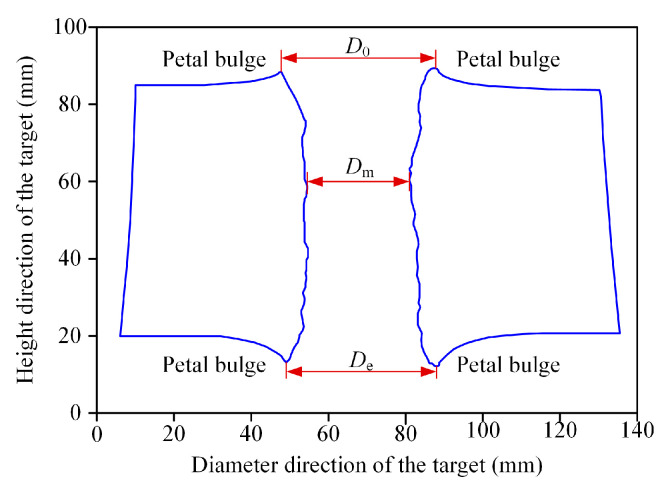
The diameter parameters of penetrated steel targets.

According to experimental results, the through-hole shape and representative crater parameters of the typical penetrated steel targets are shown in Figure 6. *D*_0_ and *D*_e_ are the diameters of the petal bulge at the front and the back of the target, respectively. *D*_m_ refers to the minimum diameter of the penetration channel, which is an important index parameter of jet perforation capabilities [26]. The crater diameter of each part is the average value calculated through three measurements to reduce errors caused by the measurement. The crater diameter of each part is the average value calculated through three measurements. The crater parameters formed on the steel targets are listed in Table 1. It can be seen that the minimum diameter of the through-hole formed due to the reactive material–Al jet was 0.46 CD. When the reactive material–Cu jet and reactive material–Ti jet penetrated the steel targets, the values of *D*_m_ were 0.32 CD and 0.41 CD, which was approximately 30.4% and 10.9% lower than that of the reactive material–Al jet, respectively.

## 3. Formation Behavior of Reactive Material Composite Jets

### 3.1. Material Model and Main Parameters

Reactive material liners can form a high-velocity jet under the detonation of the SC, and its penetration and deflagration coupling effect on the target is very complex. At present, there is no material model mature enough for the numerical simulation of the whole process of the jet forming, penetration and deflagration coupling of the SC with reactive material liners. For this reason, the reaction delay time of reactive materials (*τ*) was introduced by some domestic and foreign scholars [17,25,27]. Generally, it has been considered that reactive material jets are inert such as traditional metal jets when time *t* < *τ*. The shock equation of state (EOS) and Johnson–Cook constitutive model were used to describe the formation and penetration behavior of the reactive material jets. Meanwhile, the corresponding parameters of the PTFE/Al reactive materials were obtained based on the separated Hopkinson shock and quasistatic compression experiments; the main material parameters are listed in Table 2 [17,28]. The material of the target was steel 45#. The EOS of the shock model incorporating the strength model of Johnson–Cook was chosen to describe the material models; the main parameters are also listed in Table 2 [17], where *ρ* is the density of the material, *a*, *b*, *n*, *C*, *m* and *S* are the material constants, *T*_m_ and *T*_room_ are the melting temperature and room temperature, respectively, *Γ* is the Gruneisen coefficient and *c*_0_ is the acoustic sound speed of the material.

For the metal liner, the shock EOSs were selected and the strength models were neglected, because the liners behaved like a fluid under the extremely large pressure and temperature during the collapse of the SC [29]. The main material parameters of the Cu liner, Ti liner and Al liner are listed in Table 3.

For the structure of the SC, the jet forming and penetration characteristics were significantly affected by the type of explosive. The JH-2 explosive was comprehensively selected considering the detonation speed, detonation pressure and operating cost. The corresponding parameters are listed in Table 4 [17], where *D* and *P*_CJ_ are the detonation speed and detonation pressure of the explosive, respectively, *A*, *B*, *R*_1_, *R*_2_ and *ω* are the material constants, *E*_0_ is the specific internal energy and *V*_0_ is the relative volume.

### 3.2. Numerical Simulation Method

**Figure 7:**
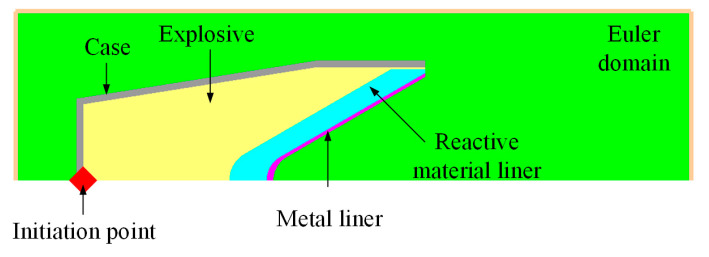
Numerical model of the jet forming for the SC with reactive material composite liner.

The formation characteristics of the SC with the reactive material composite liners were studied based on the AUTODYN-2D code, and the numerical model is shown in Figure 7. The parts of the SC, including the explosive, composite liner and case, were all simulated using a Euler method. According to the mesh sensitivity of the reference [30], the size of the Euler domain was set to 0.25 mm × 0.25 mm. In addition, the boundary condition of the model was the flow-out. The initiation point was located at the center of the bottom of the SC.

### 3.3. Formation Characteristics of Composite Jets

**Figure 8:**
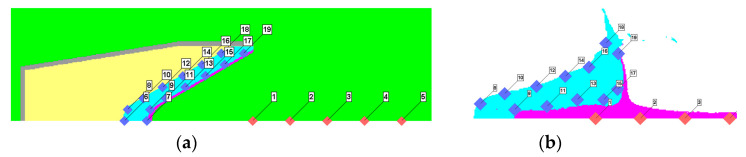
Gauge position settings: (**a**) gauges on the reactive material liner and axis of the SC; (**b**) gauges on the reactive material composite jet.

A series of gauge points were set on the outer and inner layers of the reactive material liners to observe the pressure and temperature evolution and distribution laws of the reactive material elements in the forming process of the composite jets, as shown in Figure 8a. When the jet head reached the standoff of 1.0 CD, a typical distribution of gauge points on the reactive material slug was generated, as shown in Figure 8b. In addition, five fixed gauge points were set on the Euler domain along the axis of the SC to record the tip velocity of the reactive material composite jets. Gauge #1 was set at the corresponding position of the bottom of the composite liner, and gauge #5 was set at the standoff of 1.0 CD.

**Figure 9:**
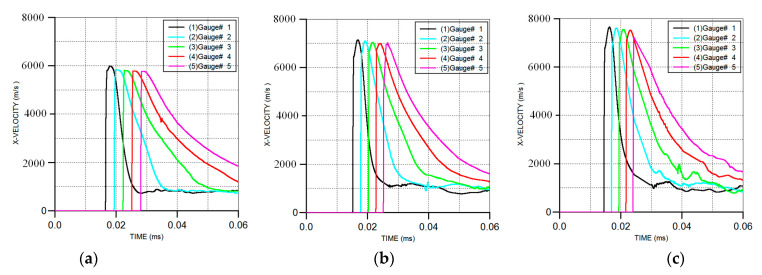
Velocity–time histories of composite jets at gauges #1~#5: (**a**) reactive material–Cu jet; (**b**) reactive material–Ti jet; (**c**) reactive material–Al jet.

**Figure 10:**
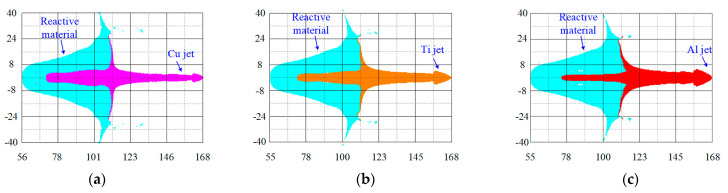
Formation morphology of composite jets before impacting the targets: (**a**) reactive material–Cu jet; (**b**) reactive material–Ti jet; (**c**) reactive material–Al jet.

**Figure 11:**
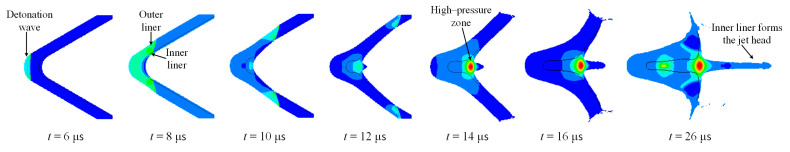
Typical detonation wave propagation and composite jet formation process.

The influence of the metal liner material on the velocity–time curves of the composite jets is shown in Figure 9. The formation morphology of the three kinds of reactive material composite jets before impacting the targets is shown in Figure 10. Although the materials of the inner metal liner were different, the morphology of the reactive material composite jets formed by the three kinds of SCs was similar. The inner metal liner mainly formed the high-velocity jet, and the outer reactive material liner became the main part of the slug. From the mechanism analysis, during the movement of the liner elements towards the axis, the detonation energy was concentrated from the outer liner to the inner liner, and the closer to the axis of symmetry, the faster the energy concentration. When moving near the axis, the elements would widen, owing to the pressure near the inner surface increasing sharply, and its movement direction would change, which finally caused the inner metal liner to become a high-velocity jet moving along the axis, as shown in Figure 11.

According to the simulation results, the performance parameters of the three kinds of reactive material composite jets are listed in Table 5, where *v*_j,1_ and *v*_j,5_ are the jet velocity at gauges #1 and #5, respectively, *d*_m,j_ is the maximum diameter of the jet head and *L*_j_ and *L*_j,m_ are the whole length of the composite jet and the length of the metal jet, respectively. The numerical results showed that *v*_j,1_ and *v*_j,5_ increased gradually as the material of the metal liner changed from Cu to Ti and Al. This was mainly because, for the SC with the same structure, the tip velocity of the jet was inversely proportional to the density of the composite liner. Compared with the reactive material–Cu jet velocity (*v*_j,5-Cu_), the jet velocity values of *v*_j,5-Ti_ and *v*_j,5-Al_ increased by 18.2% and 25.1%, respectively. In addition, by comparing the velocity of the reactive material–Ti jet with that of the reactive material–Al jet, *v*_j,1-Al_ was approximately 7.2% higher than *v*_j,1-Ti_, while *v*_j,5-Al_ was only approximately 5.8% higher than *v*_j,5-Ti_. This phenomenon showed that the tip velocity of the reactive material–Al jet was greatly affected by the standoff. Specifically, when the standoff was 1.0 CD, the *v*_j,5-Al_ decreased by approximately 5.7% compared with *v*_j,1-Al_.

As to the diameter of the composite jet before impacting the target, the diameter of the reactive material–Cu jet (*d*_m,j-Cu_) was the smallest, and the diameter of the reactive material–Al jet (*d*_m,j-Al_) was the largest. The value of *d*_m,j-Al_ was 10 mm, which was approximately 85.2% and 25% larger than *d*_m,j-Cu_ and *d*_m,j-Ti_, respectively. In terms of the length of the composite jet, the total length of the three composite jets was almost the same. However, the length of the metal jet would drop off gradually with the decrease in the material density of the inner liner. That is, the value of *L*_j,m-Al_ was the smallest, which was approximately 9.9% and 5.8% smaller than *L*_j,m-Cu_ and *L*_j,m-Ti_, respectively.

**Figure 12:**
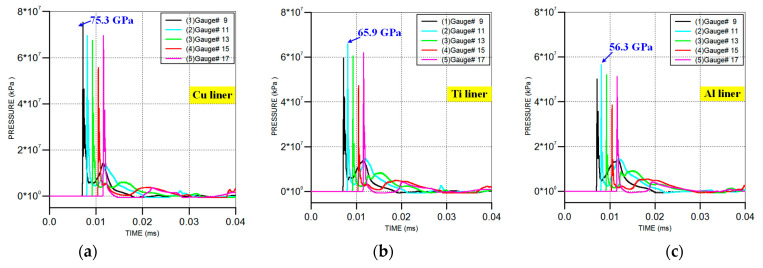
Pressure–time history curves of gauge points during the composite jet formation process: (**a**) reactive material–Cu jet; (**b**) reactive material–Ti jet; (**c**) reactive material–Al jet.

Figure 11 also shows that the detonation wave spread gradually from the outer layer of the reactive material liner to the inner layer of the metal liner during the process of jet forming. When the jet head formed (*t* = 14 µs), a high-pressure zone was produced at the interface between the metal jet and the slug. As the jet head continued to go forward, the high-pressure zone gradually moved to the interface between the two materials. The pressure–time curves of the gauges in the reactive material liner are shown in Figure 12. It can be seen that there were two pressure peaks at each gauge point. At *t* = 7µs, the pressure reached a peak at gauge #9, followed by gauge points 11, 13, 15 and 17. As the material of the metal liner changed from Cu to Ti and Al, the instantaneous peak pressure at the gauge points decreased from 75.3 GPa to 65.9 GPa and 56.3 GPa. At *t* = 13µs, the second pressure peak appeared at gauges #9 and #11. However, compared with the first pressure peak, the second peak was significantly lower. Combining Figure 11, it can be seen that the detonation wave transmitted from the outer layer of the reactive liner to the inner elements, and from the top to the bottom of the liner. As such, the first pressure peak was generated by the explosive detonation wave, while the second peak was generated by the collision between the inner material elements of the reactive liner and the metal ones. The pressure generated by the collision was much lower than that of the explosion. As the jet forming entered a stable state, the pressure at these gauge points gradually decreased and tended to fall to zero.

**Figure 13:**
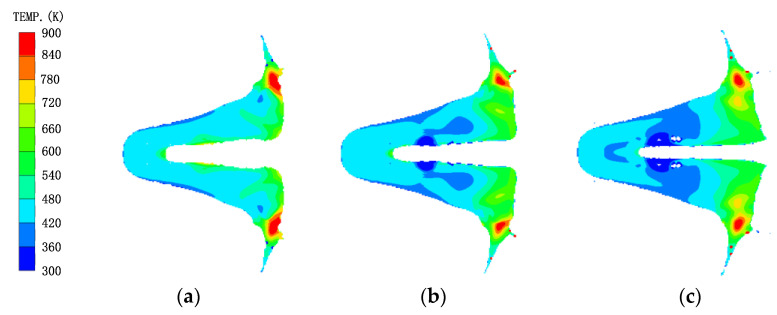
Temperature distribution of the reactive material slug under different composite jets: (**a**) reactive material–Cu jet; (**b**) reactive material–Ti jet; (**c**) reactive material–Al jet.

**Figure 14:**
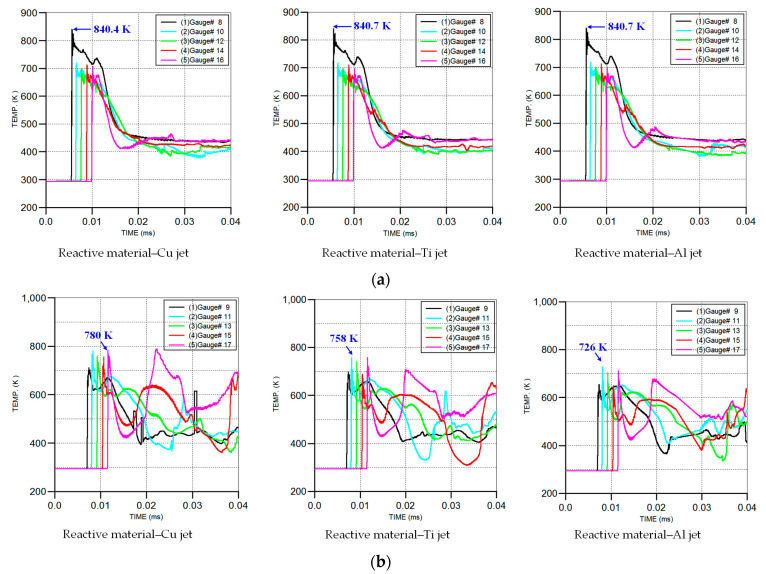
Temperature–time history curves of gauge points in the reactive material liner during the jet formation process: (**a**) gauge points on the outer layer; (**b**) gauge points on the inner layer.

Under the effect of the high-pressure detonation wave, the reactive material liner was be crushed and gradually formed the main part of the slug. The detonation wave propagation and the plastic deformation of the reactive material liner would cause the temperature rise effect [20]. When the jet head reached the standoff of 1.0 CD, the temperature distribution of the slug formed by the reactive material liner is shown in Figure 13. The temperature–time curves of the gauge point settings on the outer and inner layers of the reactive material liner are shown in Figure 14.

Figure 14a shows that the temperature peak first appeared at gauge #8 on the outer layer of the reactive liner, and then at gauge points 10, 12, 14 and 16. Under the three conditions, the peak temperature at gauge #8 was approximately 840 K, and there was little difference between the peak temperature at other gauge points. This was mainly because of the same structure of the shaped charges; that is, the outer layer elements of the reactive liner were subjected to the same explosive detonation wave, resulting in almost no difference in the temperature rise of these elements. Compared with Figure 14a, the temperature peaks at several gauge points in the inner layer of the reactive liner in Figure 14b occurred two or more times. This was mainly because the temperature rise in the inner elements of the reactive liner was mainly composed of three parts: one was the temperature rise caused by the explosive detonation wave, the second was that caused by the plastic deformation and the third was that caused by the collision and extrusion between the inner elements of the reactive liner and the metal liner elements. When the metal liner changed from Cu to Ti and Al, the instantaneous peak temperature of the gauges decreased from 780 K to 758 K and 726 K, respectively.

Meanwhile, Figure 13 also shows that the temperature of the majority of the reactive material elements was below 700 K, while only some elements at the two wings of the slug were more than 900 K. In fact, the material elements of the PTFE/Al liner could be activated under the detonation wave of the SC, but only when the temperature of the PTFE-based rose to approximately 900 K and decomposed enough oxidant C_2_F_4_ could the Al particles experience a violent detonation-like reaction with the released oxidant [12,31]. As such, the elements at the two wings of the slug may have had a chemical reaction during the process of jet forming and penetration. However, it was difficult to cause the overall reaction of the reactive materials due to their small mass.

These phenomena further verified that the chemical reaction of the PTFE/Al liner material was negligible when time *t* < *τ*. The time interval between the initiation of the SC and the violent detonation-like reaction is generally called the reaction delay time, and the chemical reaction behavior can be ignored in the time range [17]. In addition, the reaction delay time *τ* was not only affected by the particle size and preparation process of the PTFE/Al liner, but also related to the impact pressure and explosive type [32]. The time *τ* could be obtained through relevant experiments. In this paper, the corresponding parameters of the PTFE/Al liner were consistent with that of the reference [17], and the reaction delay time *τ* was approximately 166.5 μs.

## 4. Perforation Mechanism of Reactive Material Composite Jet

### 4.1. Perforation Behavior of Composite Jets Due to Its Kinetic Energy

**Figure 15:**
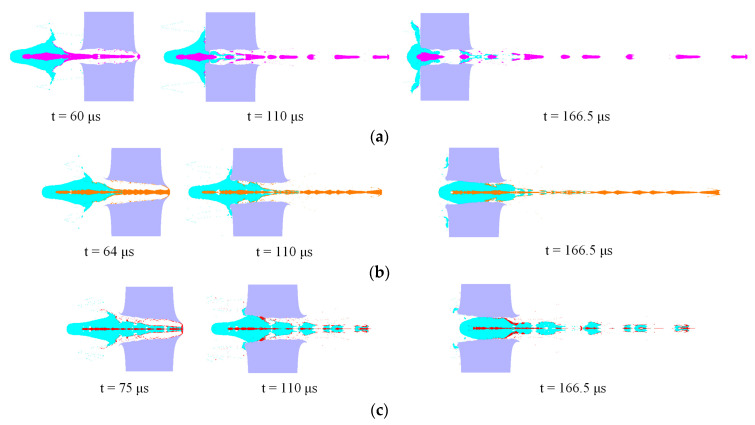
Penetration process of reactive material composite jets against steel targets: (**a**) reactive material–Cu jet; (**b**) reactive material–Ti jet; (**c**) reactive material–Al jet.

For time *t* ≤ *τ*, the penetration processes of the three kinds of composite jets due to their kinetic energy are shown in Figure 15. The numerical results showed that the 1.0 CD thick steel targets were all perforated with the three kinds of reactive material composite jets, causing penetration holes with different diameters to be formed.

Figure 15a,b demonstrate that the reactive material–Cu jet and reactive material–Ti jet penetrated the steel target through the high-velocity metal jets, while Figure 15c shows that the Al jet section was consumed before the reactive material–Al jet perforated the target. From the penetration theory of the SC jet [33], the penetration depth was proportional to the effective length of the jet. When the standoff was 1.0 CD, Table 6 shows that the length of the metal Cu jet and Ti jet was larger than that of the Al jet. At the same time, the penetration depth was also proportional to the square root of the ratio of the jet and the target density. This showed that the higher the density of the metal jet at the head, the greater would the penetration depth of the same material target be. In other words, when penetrating a steel target with the same thickness and material, a jet with the smaller density would be consumed more seriously.

Figure 15 also shows that the type of inner metal liner had a significant influence on the reactive material’s distribution. For the reactive material–Cu jet, when the penetration time reached the initiation delay time *τ*, most of reactive materials were blocked outside the entrance of the penetration hole. This was mainly due to the smaller diameter and lower velocity of the reactive material–Cu jet head (seen in Table 5), resulting in a smaller crater diameter on the entrance of the steel target. In terms of the mechanism, the hole diameter of the penetrated target was proportional to the head diameter and tip velocity of the SC jet based on the radial crater growth theory [34,35]. For the reactive material–Ti jet and reactive material–Al jet, Figure 15b,c show that almost all of the reactive materials could enter into the penetration hole, and the great mass of the reactive materials was right in the perforation channels. Furthermore, compared with the reactive material–Ti jet, more reactive materials could go through the penetration channel with the reactive material–Al jet impact, and the distribution behind the target was relatively uniform at each position.

**Figure 16:**
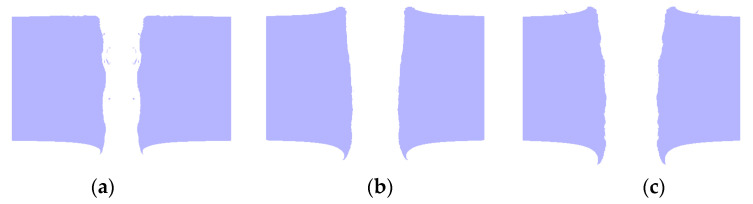
Numerical results of kinetic energy penetration for the three kinds of composite jets: (**a**) reactive material–Cu jet; (**b**) reactive material–Ti jet; (**c**) reactive material–Al jet.

Under the three conditions, the penetration results of the steel targets through the only kinetic energy of the reactive material composite jets are shown in Figure 16. The penetration hole diameters (*D*_0,p_, *D*_m,p_ and *D*_e,p_) formed on the steel targets and the errors between the numerical and experimental results are listed in Table 6. It can be seen from Table 6 that under the action of the only kinetic energy penetration of the reactive material composite jets, the different hole diameters were greatly smaller than that of the experimental results. The minimum difference between the numerical simulations and experimental results was 8.9%, and the maximum difference was 13.2%.

### 4.2. Re-Expansion Cratering due to Detonation-like Effects of Reactive Materials

According to the references [36,37], it is considered that the PTFE/Al reactive materials can have a violent detonation-like reaction instantly at *t* ≥ *τ*, and the behavior can be described through the EOS of the JWL. The corresponding parameters of the reactive material detonation-like effects are listed in Table 7.

**Figure 17:**
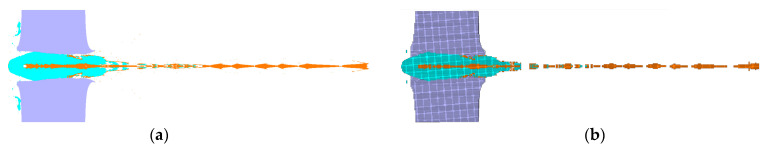
Algorithm transformation and simulation method of the detonation-like reaction effects: (**a**) 2D model at τ; (**b**) 3D SPH model.

Based on the discussion above, the penetration process of the reactive material composite liner shaped charge against the steel target was divided into two stages. The first stage was the process of the composite jet forming and penetrating the steel target. The second stage was the violent detonation-like reaction of the PTFE/Al reactive materials. At the second stage, it was necessary to save the effective mesh data of the composite jets and steel targets in a two-dimensional Euler–Lagrange domain at time *τ*, as shown in Figure 17a. On this basis, a 3D model was established based on the SPH algorithm. Then, the extracted 2D model was transformed into a 3D SPH model, as shown in Figure 17b. At the same time, the JWL model of the reactive materials was added, which would simulate the damage enhancement behavior caused by the chemical reactions of the PTFE and Al powders.

**Figure 18:**
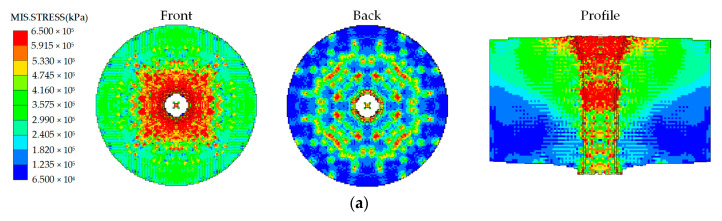

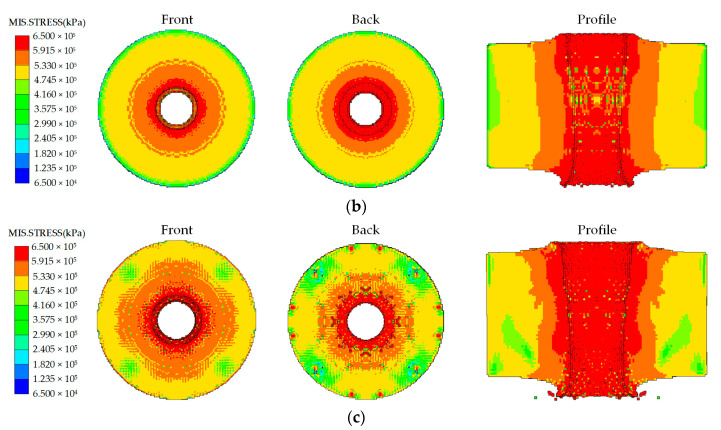
Combined perforation effects of composite jets against steel targets after the detonation-like reaction of reactive materials: (**a**) reactive material–Cu jet; (**b**) reactive material–Ti jet; (**c**) reactive material–Al jet.

The combined perforation effects of the reactive material composite jets against the steel targets after the detonation-like reaction of the reactive materials are shown in Figure 18. The hole diameters of the steel targets produced due to the detonation-like effects of the reactive materials (*D*_0,d_, *D*_m,d_ and *D*_e,d_) and the errors between the numerical and experimental results are listed in Table 8. It can be seen from Table 8 that the simulated hole diameters with the detonation-like reaction of the PTFE/Al reactive materials were similar to that of the experimental results, and the difference between them was within 5%. In fact, the detonation-like reaction of the PTFE/Al reactive materials would release a large amount of chemical energy and gas products, i.e., AlF_3_, AlF_2_ and AlF [12,14,17], which would produce overpressure in the penetration channel and further expand the initial crater size. Therefore, the coupling simulation method used the EOS of shock, the Johnson–Cook model and the JWL equation to describe the penetration and detonation-like reaction behavior of the PTFE/Al reactive materials, showing a good computational accuracy.

## 5. Conclusions

In this paper, reactive material composite liners were fabricated with machining, cold pressing and sintering processes. On this basis, the perforation behavior of reactive material composite liners shaped charges against steel targets were investigated using numerical simulations and static experiments. Several conclusions were presented as follows:

(a) The experimental results showed that when the thickness of the steel target was 1.0 CD, the penetration hole diameters of the reactive material–Al jet and reactive material–Ti jet were significantly larger than that of the reactive material–Cu jet. In particular, the entrance hole and minimum diameters formed due to the reactive material -Cu jet were approximately 26.8% and 30.4% smaller than that of the reactive material–Al jet.
(b) For the structure of the composite liner shaped charges, the inner metal liner material had a little influence on the jet morphology. However, it significantly affected the tip velocity and head diameter of the reactive material composite jet. As the material of the metal liner changed from Cu to Ti and Al, the jet tip velocity and diameter increased gradually, which was an important reason for the larger hole diameter formed by the reactive material–Al jet.
(c) The hole diameters of the three kinds of reactive material composite jets obtained through numerical simulations were in good agreement with the corresponding experimental results. This proved that the simulation method combining the inert assumption during the jet formation and penetration and the JWL equation describing the detonation-like reaction behavior of the PTFE/Al reactive materials was reliable.

## Figures and Tables

**Table 1:** Experimental results of penetration hole diameters under different reactive material composite jets against steel targets.

NO.	Composite Jet	*D*_0_ (CD)	*D*_m_ (CD)	*D*_e_ (CD)
1	Reactive material–Cu jet	0.52	0.32	0.44
2	Reactive material–Ti jet	0.64	0.41	0.57
3	Reactive material–Al jet	0.71	0.46	0.61

**Table 2:** Main parameters of the unreacted PTFE/Al reactive materials and steel target.

Materials	*ρ* (kg/m^3^)	*G* (GPa)	*a* (MPa)	*b* (MPa)	*n*	*C*	*m*	*T*_m_ (K)	*T*_room_ (K)	*Γ*	*c*_0_ (m/s)	*S*
PTFE/Al	2.27	0.67	8.04	250.6	1.8	0.4	1.03	500	294	0.9	1450	2.26
Steel 45#	7.83	77	507	320	0.28	0.064	1.06	1793	300	2.17	4570	1.49

**Table 3:** Main parameters of Cu, Ti and Al liner materials.

Materials	*ρ* (kg/m^3^)	*Γ*	*c*_0_ (m/s)	*S*
Cu liner	8.93	2.02	3940	1.489
Ti liner	4.53	1.09	5220	0.767
Al liner	2.78	2	5328	1.338

**Table 4:** Material parameters of the JH-2 explosive.

Material	*ρ* (g/cm^3^)	*D* (km/s)	*P*_CJ_ (GPa)	*A* (GPa)	*B* (GPa)	*R*_1_	*R*_2_	ω	E_0_ (GPa)	V_0_
Explosive	1.71	8.315	28.6	524.23	7.678	4.2	1.1	0.34	8.499	1.00

**Table 5:** Performance parameters of the three kinds of reactive material composite jets.

Materials	*v*_j,1_ (m/s)	*v*_j,5_ (m/s)	*d*_m,j_ (mm)	*L*_j_ (mm)	*L*_j,m_ (mm)
Reactive material–Cu jet	5985	5768	5.4	112	59.3
Reactive material–Ti jet	7142	6820	8.0	113	56.7
Reactive material–Al jet	7653	7218	10.0	113	53.4

**Table 6:** Comparison between numerical penetration hole diameters and experimental results.

Composite Jet	Comparison *D*_0_		Comparison *D*_m_		Comparison *D*_e_	
	*D*_0,p_ (mm)	Errors	*D*_m,p_ (mm)	Errors	*D*_e,p_ (mm)	Errors
Reactive material–Cu jet	30.2	−13.2%	18.8	−10.5%	26.6	−8.9%
Reactive material–Ti jet	38.6	−9.0%	24.0	−10.5%	34.0	−9.1%
Reactive material–Al jet	40.6	−13.2%	27.2	−11.5%	36.0	−10.4%

**Table 7:** Main parameters of the reacted PTFE/Al reactive materials.

Materials	*D* (km/s)	*P*_CJ_ (GPa)	*A* (GPa)	*B* (GPa)	*R_1_*	*R_2_*	*ω*
PTFE/Al	5.200	0.67	15.9	0.0023	7	0.6	0.38

**Table 8:** Comparison between experimental results and numerical penetration hole diameters after the detonation-like reaction of reactive materials.

Composite Jet	Comparison *D*_0_		Comparison *D*_m_		Comparison *D*_e_	
	*D*_0,d_ (mm)	Errors	*D*_m,d_ (mm)	Errors	*D*_e,d_ (mm)	Errors
Reactive material–Cu jet	33.6	−3.4%	20.2	−3.8%	28.4	−2.7%
Reactive material–Ti jet	42.0	−0.7%	27.2	+1.5%	36.8	−1.6%
Reactive material–Al jet	47.8	+2.1%	29.4	−3.6%	41.6	+3.5%

## Data Availability

Not applicable.
